# Short term variation in NTproBNP after lifestyle intervention in severe obesity

**DOI:** 10.1371/journal.pone.0181212

**Published:** 2017-07-13

**Authors:** Debora Fedele, Virginia Bicchiega, Alessandro Collo, Federica Barutta, Erika Pistone, Gabriella Gruden, Graziella Bruno

**Affiliations:** 1 Department of Medical Sciences, University of Turin, Turin, Italy; 2 Italian Auxologic Institute, Verbania, Italy; Ospedale del Cuore G Pasquinucci Fondazione Toscana Gabriele Monasterio di Massa, ITALY

## Abstract

**Aim:**

Natriuretic peptides are not only involved in cardiovascular adaption to various conditions, but also in metabolic diseases. We performed this study to assess the effect of a very short time of lifestyle inpatient intervention on NTproBNP values in normotensive subjects with severe obesity and normal cardiac function.

**Methods:**

We recruited 14 consecutive obese normotensive subjects with normal cardiac function who were aged 30 years and more and were referred to inpatient rehabilitation in an academic clinic over a two months period. They were examined at baseline and after a 3-weeks program including dietary intervention with hypocaloric diet and assisted personalized physical aerobic and anaerobic activities and compared to age, sex and BMI-matched control subjects under usual care.

**Results:**

BMI significantly decreased (40.8 ±1.6 vs 42.3 ± 1.6 kg/m^2^, p <0.0001). Median reduction in body weight was 4.9 kg (interquartile range 2.4–5.2 kg). After diet and exercise-induced weight loss, plasma NTproBNP levels showed an almost two-fold increase, which was statistically significant (28.2 ± 12.3 vs 17.2 ± 13.2 ng/L, p = 0.01), and particularly relevant in the subgroup with NT-proBNP values below median values compared to those with higher values (p = 0.02). No significant variations were found in control subjects (18.0 ± 13.0 vs 16.5 ± 11.2 ng/L, p = 0.18). The lipid profile was significantly ameliorated, and both HbA1c and insulin levels showed a marginally non-significant decrease after treatment.

**Conclusions:**

An almost two-fold increase in NTproBNP levels was evident after a very short time period of lifestyle intervention in normotensive severe obese patients without cardiac disease. This finding might have clinical relevance, considering the role of NT-proBNP as risk factor of impaired glucose tolerance.

## Introduction

B-type natriuretic peptide (BNP) belongs to the family of the natriuretic peptides [1]. Stress of the ventricular wall due to volume and/or pressure overload is the main inducer of BNP transcription [1] and BNP plays a key role in cardiovascular homeostasis, counteracting the deleterious effects of cardiac overload and exerting anti-fibrotic and anti-hypertrophic effects in the heart [2]. The active peptide BNP is released in equimolar concentrations with the inactive fragments NT-proBNP, which has a longer plasma half-life, and both BNP and NT-proBNP are well-established diagnostic/prognostic biomarkers of heart failure [3] and independent predictors of cardiovascular mortality [4–6].

Obesity is associated with cardiovascular diseases; however, BNP and NT-proBNP levels are paradoxically reduced in obese subjects and a negative linear relationship between body mass index (BMI) and both BNP and NT-proBNP levels has been consistently reported in epidemiological studies [7–9]. Recently, this inverse association has been also confirmed in morbid obese subjects [10]. In addition, low levels of NT-proBNP are a strong predictor of type 2 diabetes onset. Indeed, a prospective cohort study has shown that lower NT-proBNP values were associated with higher risk of incident type diabetes over a 12-years follow-up period, independently of confounders and risk factors [11] and a common genetic BNP variant (rs198389), which results in a 20% increase in plasma BNP levels, was found associated with a 15% reduced risk of diabetes [12]. The underlying cause of the relative BNP deficiency in obese insulin-resistant subjects (natriuretic handicap) is not entirely known, but both a reduced synthesis/release from the heart and an enhance clearance thought the type C natriuretic peptide receptor have been implicated [13–15]. Regardless of the mechanism, this natriuretic handicap is of high clinical relevance as it may expose obese patients to enhanced cardiovascular risk. Moreover, there is emerging evidence that natriuretic peptides play a direct role in the control of metabolic processes by enhancing lipolysis and energy expenditure [13,16]. Therefore, low BNP levels may be not only a consequence, but also a cause/contributing factor in obesity.

There is relatively little information on the effect of body weight loss on circulating NT-proBNP levels. After gastric bypass surgery, BNP was reported to increase and to correlate with weight reduction [17,18]. Data on the effect of weight loss following lifestyle intervention are more conflicting and both increased and reduced NT-proBNP levels have been reported [19–21]. Therefore, it is of interest to assess whether natriuretic peptide levels may vary after a short-term dietary and physical program in obese people without other comorbidities. In this study, we assessed the effect of a short-time lifestyle inpatient intervention on NT-proBNP values in normotensive subjects with severe obesity and normal cardiac function.

## Materials and methods

The study recruited 14 consecutive subjects aged ≥30 years with body mass index (BMI)≥ 30 kg/m^2^ who were referred over a two months period to the Italian Auxologic Institute (Verbania, Italy) for inpatient rehabilitation. Patients were excluded if they had hypertension, diabetes, chronic heart failure, cardiac valvulopathy, cirrhosis, chronic obstructive pulmonary disease, cancer and estimated glomerular renal function < 60 ml/min/1.73m^2^. All subjects were examined at baseline and after a 3-weeks inpatient program including a dietary intervention with hypocaloric diet, and assisted personalized physical activities (aerobic and anaerobic). We also examined an age (±5 years), sex and BMI (±5 kg/m^2^) matched control group of 14 subjects who were referred to the outservice clinic for severe obesity of the Turin University Hospital, both at the first referral visit and after 3-weeks. They received written dietary advice by a dietitian at the baseline visit only. All individuals gave written informed consent and the study was carried out in accordance with the Declaration of Helsinki. The study was approved by the Ethical Committee of Turin University. Anthropometric measurements (BMI, waist circumpherence and body composition) were assessed using standardized methods by two medical researchers. Fat mass and fat-free mass were determined by bioelectrical impedance (BIA 101, Akern, Florence, Italy). Blood pressure was measured using an aeroid sphygmomanometer. All laboratory measurements were centralized. Venous blood samples were collected after at 8.00 a.m. after overnight fasting and at least for 20 min resting in supine position for determination of creatinine, glycaemia, insulin, total and HDL-cholesterol, triglycerides and uric acid. LDL-cholesterol was calculated using the Friedewald’s formula. An electro-chemiluminescent immunoassay (ECLIA, Roche Diagnostics International, Rotkreuz, Switzerland) was used to measure levels. All patients performed at baseline ECG and a M-mode two-dimensional echocardiography (Sonos 2500; Hewlett-Packard) to recruit only asymptomatic subjects with normal cardiac rhythm and left ventricular systolic function.

Variables distributed normally are presented as mean and standard deviation (SD). Comparisons were performed using the paired Student t test and the χ^2^ test as appropriate. The sample size provided a power of 92% (a = 0.05) to detect a difference in NT-proBNP of at least 85% of a standard deviation between baseline to the follow-up examinations. Linear linear regression was performed to assess the association between NTproBNP, BMI and weight reduction. All P values were 2-sided, and P values of less than 0.05 were considered statistically significant. STATA version 10.0 was used for the analyses.

## Results

The study group (n = 14) had a mean age of 37.4 (± 12.1) years, a predominance of women (M/F 4/10), and various grade of obesity with a BMI ranging from 34.1 to 55.0 kg/m^2^. All patients were asymptomatic and left ventricular systolic function was normal (ejection fraction 61.0 ± 4.5%). As shown in Table 1, the intervention was effective in reducing body weight [median body weight reduction: 4.9 kg (interquartile range 2.4–5.2 kg)], BMI, and waist circumference. There were no significant changes in body composition and blood pressure, while lipid profile was significantly ameliorated. Both HbA1c and insulin levels showed a marginally non-significant decrease after treatment.

**Table 1:** Anthropometric and metabolic values at baseline and after a 3 weeks period treatment.

	Baseline	After 3 weeks	Δ	P
**Body weight (kg)**	118.6 ± 24.1	114.5 ± 23.8	4.1 ± 1.5	<0.0001
**BMI (kg/m**^**2**^**)**	42.3 ± 1.6	40.8 ± 1.6	1.5 ± 0.6	<0.0001
**Waist circumference (cm)**	122.8 ± 14.1	114.9 ± 15.9	7.8 ± 5.5	0.0002
**Systolic blood pressure (mmHg)**	119.3 ± 3.8	118.6 ± 3.4	___	0.83
**Diastolic blood pressure (mmHg)**	74.3 ± 3.7	77.8 ± 3.0	___	0.39
**Fat mass (%)**	51.3 ± 1.9	52.2 ± 1.6	___	0.34
**Fat-free mass (%)**	48.8 ± 7.6	48.4 ± 7.5	___	0.71
**Glucose (mg/dl)**	96.4 ± 7.0	86.8 ± 2.7	___	0.19
**HbA1c (%)**	5.5 ± 1.0	5.2 ± 0.6	___	0.06
**Insulin (mIU/L)**	18.6 ± 6.3	14 ± 5.1	___	0.06
**Total cholesterol (mg/dl)**	191.1 ± 45.3	165.1 ± 45.3	26 ± 21.5	0.0006
**LDL-cholesterol (mg/dl)**	130.2 ± 37.1	112.3 ± 38.2	17.9 ± 18.1	0.006
**HDL-cholesterol (mg/dl)**	44.4 ± 12.3	50.2 ± 15	5.8 ± 6.8	0.007
**Triglycerides (mg/dl)**	122.4 ± 56.2	102.8 ± 46.3	___	0.07
**Uric acid (mg/dl)**	5.7 ± 1.1	5.9 ± 1.2	___	0.06

**Fig 1:**
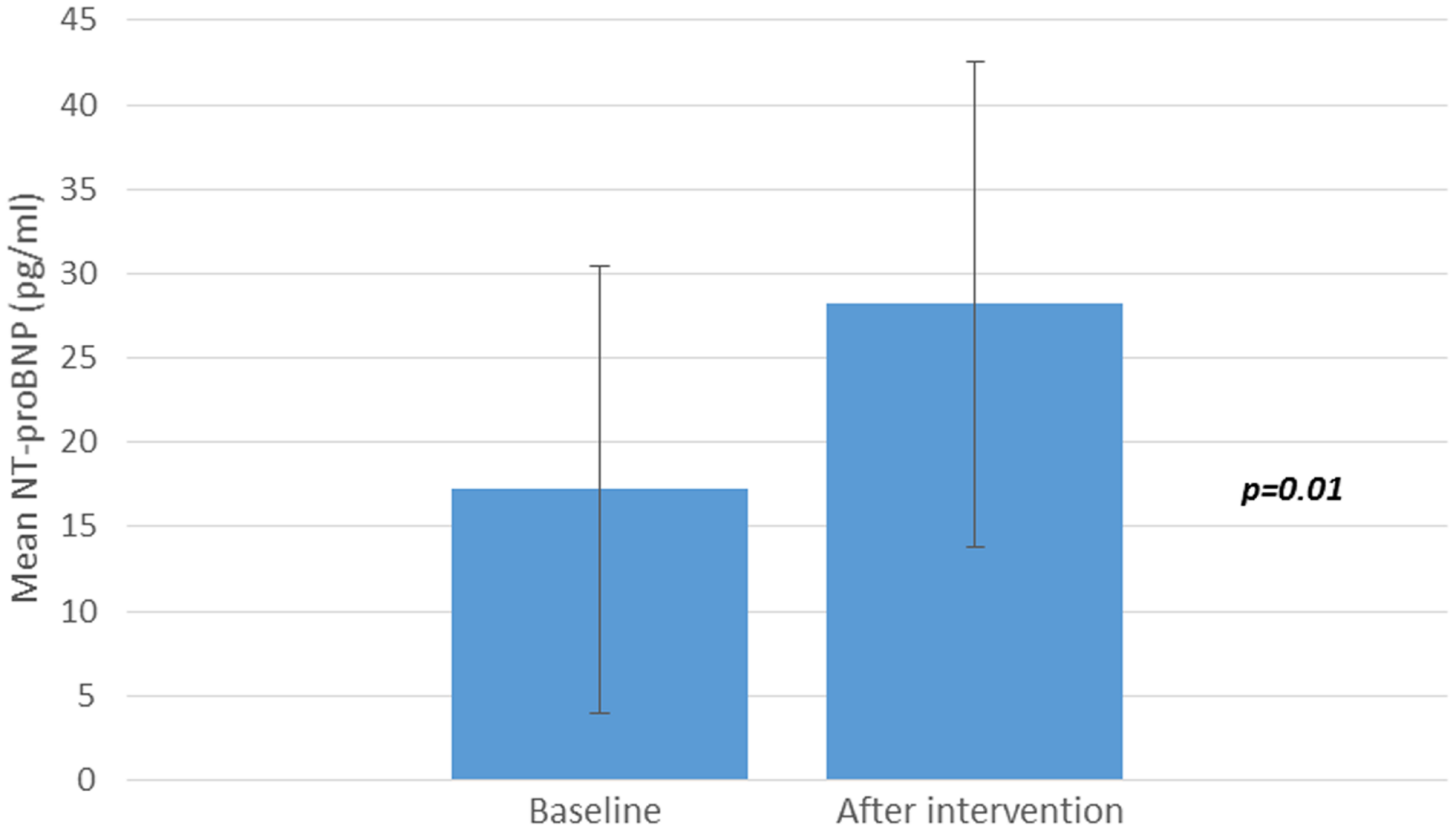
NTproBNP plasma values (ng/L) at baseline and after a 3 weeks period treatment.

Baseline mean plasma NT-proBNP levels were 17.2 ± 13.2 ng/l. After diet and exercise-induced weight loss, there was a significant twofold increase in plasma NT-proBNP levels (28.2 ± 14.4 vs 17.2 ± 13.2 ng/L, p = 0.01) (Fig 1).

The increase was particularly relevant in people who had NT-proBNP levels at or below the detection level of 5 ng/L (n = 5). Compared to obese people who had NTproBNP levels above the median value (13.0 ng/L), those who had lower levels showed the highest increase after lifestyle intervention (19.1 ± 14.1 ng/L vs 2.9 ± 8.5 ng/L, p = 0.02).

In the control group (n = 14, BMI = 122.3 ± 18.3 kg/m^2^), no significant variations in NT-proBNP levels were found over time (18.0 ± 13.0 vs 16.5 ± 11.2 ng/L, p = 0.18).

In linear regression, NTproBNP and BMI after weight loss were not significantly associated (β = 1.01, p = 0.11). Similarly, no association was evident between NT-proBNP values measured at baseline and after weight loss (β = 0.31, p = 0.24) and between Δ weight and ΔNT-proBNP (β = -2.13, p = 0.43).

## Discussion

This study provides the original evidence that weight reduction after a short period of lifestyle intervention in normotensive severe obese patients without cardiac diseases and other comorbidities was associated with an almost two-fold significant increase in circulating NT-proBNP levels. This finding is clinically relevant in the light of recent evidence that low levels of natriuretic peptides are strong independent predictors of progression from normal to impaired glucose tolerance [11–12]. In this context, results of our study, although limited by the cross-sectional study design, suggest that the increase in NT-proBNP plasma levels might be a marker of a parallel reduction in metabolic risk of these subjects. Indeed, a parallel tendency towards improvement in plasma levels of Hba1c, insulin and lipids was also found, due to an almost 5 kg weight reduction obtained during a 3-weeks of intensive treatment in the setting of a regional specialized clinic devoted to severe obese subjects. However, only prospective studies assessing incidence of cardiovascular mortality in people with increased values of NT-proBNP after lifestyle intervention might provide a definite conclusion on this issue.

Our finding of a short-term temporal relationship between NT-proBNP plasma levels and weight reduction is consistent with previous studies examining the sustained association between weight loss and natriuretic peptides in different contexts. In a prospective cohort of 131 obese subjects who underwent a three-months lifestyle intervention, significant increases in BNP were found and change in BMI were related to change in BNP [17]. The study, however, recruited an heterogeneous group of high-risk obese subjects, having either coronary heart disease or three and more risk factors, including diabetes, and results might have been biased by confounders. By contrast, in our study inclusion criteria were very stringent and only asymptomatic normotensive obese patients without other comorbidities, such as diabetes and cardiopulmonary diseases, were included. Another study confirmed the effect of changes in body weight with one year dietary intervention on natriuretic peptides [18]. By contrast, a small study found decreased concentrations of natriuretic peptides after hypocaloric diet in obese patients with essential hypertension [22].

Two other studies examined the sustained effect of weight decrease obtained through bariatric surgery on NT-proBNP levels [18–19]. In the first one, an increase in NT-proBNP values was evident in 132 obese subjects at two postoperative time point (2 and 6 months), with a 3.4 and 5-fold increase in NT-proBNP levels at 12-months [18]. Similar results were found in the second study, recruiting 98 obese subjects who underwent gastric bypass surgery, with an increase in BNP levels at each postoperative time point (3, 6, 9 months) compared with preoperative values, up to a three-fold increase after one year [19]. Interestingly, in a one-year non-randomized controlled clinical trial, the increase in NT-proBNP was higher in obese subjects who underwent gastric bypass surgery compared to obese subjects treated with lifestyle intervention, and correlated significantly with changes in body weight [21].

In no previous study the increase in NT-proBNP was attributable to conditions that typically upregulate NT-proBNP secretion, such as elevated blood pressure, cardiac hypertrophy, increased left ventricular filling pressures or ventricular dysfunction, whereas evidence is emerging on the metabolic roles played by natriuretic peptides in obesity and diabetes development [13]. Indeed, the cardiac endocrine response is the integrated resultant of several pathophysiological interactions, which are even more complex in obese subjects and not yet fully elucidated [14]. In spite of the cardiac overload, NT-proBNP and BNP levels are paradoxically reduced in obese subjects and relative natriuretic peptide deficiency has been hypothesized to be involved in the increased cardiovascular risk of obese subjects (the natriuretic handicap) [10,14, 22]. Adipose tissue expansion, a hallmark of obesity, is linked to chronic low-grade inflammation and increased secretion of adipokines which increase insulin-resistance [13]. On the other hand, hyperinsulinemia may contribute to lower levels of natriuretic peptides by up-regulating natriuretic peptide clearance receptor (NPRC) expression in subcutaneous fat tissue [23]. Adipose tissue lipolysis is strongly regulated by insulin (antilipolytic) and catecholamines (lipolytic). More recently, it has been found that natriuretic peptides, including BNP, have lipolytic activity also [13, 24]. Visceral fat expansion can increase the clearance of active natriuretic peptides by means of an increased expression of clearance receptors on adipocytes, and in this way, it may contribute to decrease the activity of the cardiac endocrine system [14]. Impaired natriuretic peptide release has also been reported with a down-regulation of NPRA mRNA and protein level in fat tissue and skeletal muscle in obesity [25]. In light of these observations, hyperglycemia and hyperinsulinemia may be the mechanisms underlying natriuretic peptides deficiency in obesity and diabetes [26].

Limitation of our study should be considered. The sample size was quite limited, but the the study was designed to provide a power of 90% and over to detect a significant variation in NT-proBNP levels over a short period of time. On the other side, we applied a wide range of exclusion criteria, which allowed to limit the effect of confounders which might have biased results, such as hypertension and cardiac hypertrophy. Cardiac function was assessed in all recruited subjects by ECG and transthoracic echocardiography, and only asymptomatic subjects with normal cardiac rhythm and ejection fraction were enrolled. However, we cannot exclude the possibility that patients with asymptomatic diastolic dysfunction were included, as this condition is frequent in obese subjects and poorly detected by M-mode two-dimensional echocardiography (15). Echocardiography post intervention was not performed, however it is unlikely that cardiac function have changed in a such short time period. Natriuretic peptides increase after exercise, however blood samples were collected after overnight fasting, and it is unlikely this might have affected our results. Finally, no subjects was treated with β-blockers-drugs, which might interfere with haemodynamic and lipolytic activities.

In conclusion, this study expands knowledge in this field showing an almost two-fold increase in NTproBNP levels after a very short period of lifestyle intervention in normotensive severe obese patients without cardiac disease, which might have further clinical implications, considering the role of NT-proBNP as risk factor of impaired glucose tolerance.

## Supporting information

S1 File The data underlying this study. (DTA)

## References

[1] Nishikimi T, Kuwahara K, Nakao K. Current biochemistry, molecular biology, and clinical relevance of natriuretic peptides. J Cardiol 2011;57:131–140 doi: 10.1016/j.jjcc.2011.01.002 21296556 10.1016/j.jjcc.2011.01.002

[2] Nishikimi T, Maeda N, Matsuoka H. The role of natriuretic peptides in cardioprotection. Cardiovasc Res 2006;69:318–328 doi: 10.1016/j.cardiores.2005.10.001 16289003 10.1016/j.cardiores.2005.10.001

[3] Kim HN, Januzzi JL Jr. Natriuretic peptide testing in heart failure. Circulation 2011;123:2015–2019 doi: 10.1161/CIRCULATIONAHA.110.979500 21555724 10.1161/CIRCULATIONAHA.110.979500

[4] Di Angelantonio E, Chowdhury R, Sarwar N, et al B-type natriuretic peptides and cardiovascular risk: systematic review and meta-analysis of 40 prospective studies. Circulation. 2009;120:2177–2187 doi: 10.1161/CIRCULATIONAHA.109.884866 19917883 10.1161/CIRCULATIONAHA.109.884866

[5] Tarnow L, Gall MA, Hansen BV, Hovind P, Parving HH. Plasma N-terminal pro-B-type natriuretic peptide and mortality in type 2 diabetes. Diabetologia 2006; 49: 2256–2262 doi: 10.1007/s00125-006-0359-4 16937127 10.1007/s00125-006-0359-4

[6] Bruno G, Landi A, Barutta F, Ghezzo G, Baldin C, Spadafora L et al NH2-terminal pro-brain natriuretic peptide is a stronger predictor of cardiovascular mortality than C-reactive protein and albumin excretion rate in elderly patients with type 2 diabetes: the Casale Monferrato population-based study. Diabetes Care 2013; 36: 2677–2682. 23564918 10.2337/dc13-0353 PMC3747916

[7] Wang TJ, Larson MG, Levy D, Benjamin EJ, Leip EP, Wilson PW et al Impact of obesity on plasma natriuretic peptide levels. Circulation 2004;109: 594–600 doi: 10.1161/01.CIR.0000112582.16683.EA 14769680 10.1161/01.CIR.0000112582.16683.EA

[8] Madamanchi C, Alhosaini H, Sumida A, Runge MS. Obesity and natriuretic peptides, BNP and NT-proBNP: mechanisms and diagnostic implications for heart failure. Int J Cardiol. 2014; 176:611–617 doi: 10.1016/j.ijcard.2014.08.007 25156856 10.1016/j.ijcard.2014.08.007 PMC4201035

[9] Sanche OA, Duprez DA, Daniels LB, Maisel AS, Otvos JD, Peralta CA et al The association between N-terminal pro B-type natriuretic peptide and lipoprotein particle concentration plateaus at higher N-terminal pro B-type natriuretic peptide values: Multi-Ethnic Study on Atherosclerosis. Metabolism 2015; 64: 857–861 doi: 10.1016/j.metabol.2015.04.001 25931335 10.1016/j.metabol.2015.04.001 PMC4782748

[10] Ricci MA, De Vuono S, Pucci G, Di Filippo F, Berisha S, Gentili A et al Determinants of low levels of brain natriuretic peptide in morbid obesity. Clin Nutr. 2016;S0261–5614 10.1016/j.clnu.2016.06.024 27426417

[11] Lazo M, Young JH, Brancati FL, Coresh J, Whelton S, Ndumele CE et al NH2-Terminal Pro-Brain Natriuretic Peptide and Risk of Diabetes. Diabetes 2013;62:3189–3193. doi: 10.2337/db13-0478 23733199 10.2337/db13-0478 PMC3749338

[12] Pfister R, Sharp S, Luben R, Welsh P, Barroso I, Salomaa V et al Mendelian randomization study of b-type natriuretic peptide and type 2 diabetes. Evidence of causal association from population studies. PLoS Med 2011; 8:e1001112 doi: 10.1371/journal.pmed.1001112 22039354 10.1371/journal.pmed.1001112 PMC3201934

[13] Gruden G, Landi A, Bruno G. Natriuretic peptides, heart, and adipose tissue: new findings and future developments for diabetes research. Diabetes Care 2014; 37:2899–908 doi: 10.2337/dc14-0669 25342830 10.2337/dc14-0669

[14] Clerico A, Giannoni A, Vittorini S, Emdin M. The paradox of low BNP levels in obesity. Heart Fail Rev 2012;17:81–96 doi: 10.1007/s10741-011-9249-z 21523383 10.1007/s10741-011-9249-z

[15] Lavie CJ, Sharma A, Alpert MA, De Schutter A, Lopez-Jimenez F, Milani RV et al Update on obesity and obesity paradox in heart failure. Prog Cardiovasc Dis 2016;58:393–400 doi: 10.1016/j.pcad.2015.12.003 26721180 10.1016/j.pcad.2015.12.003

[16] Collins S. A heart-adipose tissue connection in the regulation of energy metabolism. Nat Rev Endocrinol 2014;10:157–163 doi: 10.1038/nrendo.2013.234 24296515 10.1038/nrendo.2013.234

[17] Chainani-Wu N, Weidner G, Purnell DM, Frenda S, Merritt-Worden T, Kemp C et al Relation of B-type natriuretic peptide levels to body mass index after comprehensive lifestyle changes. Am J Cardiol. 2010;105:1570–1576 doi: 10.1016/j.amjcard.2010.01.016 20494664 10.1016/j.amjcard.2010.01.016

[18] Kistorp C, Bliddal H, Goetze JP, Christensen R, Faber J. Cardiac natriuretic peptides in plasma increase after dietary induced weight loss in obesity. BMC Obes. 2014; 1: 24 doi: 10.1186/s40608-014-0024-2 26217511 10.1186/s40608-014-0024-2 PMC4511261

[19] Chen-Tournoux A, Khan AM, Baggish AL, Castro VM, Semigran MJ, McCabe EL et al Effect of weight loss after weight loss surgery on plasma N-terminal pro-B-type natriuretic peptide levels. Am J Cardiol 2010;106:1450–1455 doi: 10.1016/j.amjcard.2010.06.076 21059435 10.1016/j.amjcard.2010.06.076 PMC3170817

[20] Changchien EM, Ahmed S, Betti F, Higa J, Kiely K, Hernandez-Boussard T et al B-type natriuretic peptide increases after gastric bypass surgery and correlates with weight loss. Surg Endosc. 2011; 25:2338–2343 doi: 10.1007/s00464-010-1565-1 21424205 10.1007/s00464-010-1565-1

[21] Gabrielsen AM, Omland T, Brokner M, Fredheim JM, Jordan J, Lehmann S et al The effect of surgical and non-surgical weight loss on N-terminal pro-B-type natriuretic peptide and its relation to obstructive sleep apnea and pulmonary function. BMC Res Notes. 2016;9:440 doi: 10.1186/s13104-016-2241-x 27619215 10.1186/s13104-016-2241-x PMC5020450

[22] Minami J, Nishikimi T, Ishimitsu T, Makino Y, Kawano Y, Takishita S et al Effect of a hypocaloric diet on adrenomedullin and natriuretic peptides in obese patients with essential hypertension. J Cardiovasc Pharmacol. 2000;36 Suppl 2:S83–6. 11206728 10.1097/00005344-200000006-00018

[23] Pivovarova O, Gögebakan Ö, Klöting N, Sparwasser A, Weickert MO, Haddad I et al Insulin up-regulates natriuretic peptide clearance receptor expression in the subcutaneous fat depot in obese subjects: a missing link between CVD risk and obesity? J Clin Endocrinol Metab. 2012;97:E731–9 doi: 10.1210/jc.2011-2839 22419733 10.1210/jc.2011-2839

[24] Moro C. Targeting cardiac natriuretic peptides in the therapy of diabetes and obesity. Expert Opin Ther Targets. 2016;20:1445–1452 doi: 10.1080/14728222.2016.1254198 27786597 10.1080/14728222.2016.1254198

[25] Coué M, Moro C. Natriuretic peptide control of energy balance and glucose homeostasis. Biochimie 2016;124:84–91 doi: 10.1016/j.biochi.2015.05.017 26037452 10.1016/j.biochi.2015.05.017

[26] Moro C. Does insulin resistance trigger natriuretic peptide deficiency? EBioMedicine. 2017;17:11–12 doi: 10.1016/j.ebiom.2017.03.005 28284816 10.1016/j.ebiom.2017.03.005 PMC5360594

